# Automatic detection of alien plant species in action camera images using the chopped picture method and the potential of citizen science

**DOI:** 10.1270/jsbbs.21062

**Published:** 2022-02-05

**Authors:** Kosuke Takaya, Yu Sasaki, Takeshi Ise

**Affiliations:** 1 Graduate School of Agriculture, Kyoto University, Kitashirakawaoiwake-cho, Sakyo-ku, Kyoto 606-8502, Japan; 2 Center for the Promotion of Interdisciplinary Education and Research, Kyoto University, Kitashirakawaoiwake-cho, Sakyo-ku, Kyoto 606-8502, Japan; 3 Field Science Education and Research Center, Kyoto University, Kitashirakawaoiwake-cho, Sakyo-ku, Kyoto 606-8502, Japan

**Keywords:** deep learning, chopped picture method, alien plant, *Solidago altissima*, action camera, citizen science, computer vision

## Abstract

Monitoring and detection of invasive alien plant species are necessary for effective management and control measures. Although efforts have been made to detect alien trees using satellite images, the detection of alien herbaceous species has been difficult. In this study, we examined the possibility of detecting non-native plants using deep learning on images captured by two action cameras. We created a model for each camera using the chopped picture method. The models were able to detect the alien plant *Solidago altissima* (tall goldenrod) and obtained an average accuracy of 89%. This study proved that it is possible to automatically detect exotic plants using inexpensive action cameras through deep learning. This advancement suggests that, in the future, citizen science may be useful for conducting distribution surveys of alien plants in a wide area at a low cost.

## Introduction

Biological invasion contributes to the decrease in biodiversity and requires measures to be taken for prevention (Levine *et al.* 2003). The impact of exotic plants on ecosystems includes competition with native species, interspecific interactions (Pauchard and Shea 2006, Rai and Singh 2020), and vegetation succession (Prach and Walker 2011). In addition, exotic plant species are fast growing and can affect soil nutrient accumulation through litter supply (Allison and Vitousek 2004); moreover, they facilitate changes in microclimate (Ruckli *et al.* 2013) and changes in fire frequency (Fusco *et al.* 2019). As it is challenging to eradicate exotic species once they are established in the field, prevention of invasion is the most effective and ideal method of control (Leung *et al.* 2002). However, a risk of invasion of alien species even while focusing on prevention, always exists. Hence, regular monitoring for early detection, and rapid responsiveness is essential (Hulme 2006).

Information on the distribution of invasive alien species is essential for effective management; however, the information is often lacking (Jarnevich *et al.* 2006). It is not practical to manually investigate a broad area, as field surveys are labor-intensive. Hence, the use of remote sensing technology has been the preferred method of surveying the distribution of alien plants. For example, previous studies employed high spatial resolution multispectral sensors to estimate leaf water content to identify exotic plants (Carter *et al.* 2009, Underwood *et al.* 2003). However, high-resolution hyperspectral data are expensive and generally limited to studies in small areas (Kganyago *et al.* 2018). Additionally, most studies that employed remote sensing techniques targeted non-native trees and shrubs; small herbaceous species are difficult to identify (Müllerová *et al.* 2017). Unmanned aerial vehicles (UAVs) have recently been used to survey exotic plants (Dash *et al.* 2019). UAVs can survey hazardous or inaccessible areas (Manfreda *et al.* 2018) and acquire high-resolution images at a low cost. However, there are areas that have banned UAVs, and thus, cannot be surveyed owing to battery limitations.

Citizen science can be a useful measure for understanding the distribution of invasive species at a large scale (Crall *et al.* 2015). It has a long history, with hobbyists and volunteers involved in the monitoring; these citizens have long been active in fields, such as astronomy and biology, where observational skills are more important than expensive equipment (Brown and Williams 2019). Science as a profession became common in the late 19th century (Silvertown 2009); until then, a considerable portion of the research was performed by non-professionals. While science has specialized further in the past 150 years, the presence of amateur scientists has declined (Miller-Rushing *et al.* 2012). However, in recent years, citizen science has been used in various fields, as information and communications technology (ICT) created an easily accessible environment for citizens (Johnson *et al.* 2020). In addition, citizen science enables large-scale data collection and provides an opportunity for interaction between citizens and nature, including improving citizens’ knowledge and changing their attitudes toward nature (Schuttler *et al.* 2018). The implementation of citizen science in ecology has also been reported worldwide and can be initiated to detect invasive species and manage their population (Maistrello *et al.* 2016, Miralles *et al.* 2016).

The growing development of citizen science is a result of the spread of observation equipment. With the increasing performance and popularity of digital cameras, small video cameras, and smartphones, it has become possible for anyone to take high-quality images anywhere. In addition, the use of platforms, such as eBird and iNaturalist simplifies the transmission of information from the field in real-time and enables ongoing citizen science projects to collect images from citizens in many places. However, although a large amount of data is easily collected, the effort required to organize the data poses a challenge. Plant identification is also challenging, thus making it difficult for non-specialists to identify plants at the species level. This creates an issue of accuracy of data (Crall *et al.* 2011, Dickinson *et al.* 2010).

Combining deep learning with citizen science may enable the survey of a wider area, with higher accuracy. Recently, deep learning is being used to identify invasive species in the field (James and Bradshaw 2020). As image recognition technology is widely used to automatically analyze large amounts of data with a certain level of accuracy, citizen science has developed further. Unlike animals, exotic plants do not move, hence making them easier to spot and photograph. In addition, exotic plants can be easily distinguished from the surrounding plants at certain times of the year, such as flowering and fall. Taking advantage of this characteristic, it is possible to target species and focus public attention on this species. In particular, flowers easily attract people’s attention and can be distinguished from other species without considerable difficulty, through their color and shape, as compared to leaves and stems. If deep learning can be used to identify alien flowers from images captured by citizens, it may become a new method for understanding the distribution of alien plants.

An increasing amount of research has been conducted on the use of deep learning methods to detect plant species from images recorded in the field (Jones 2020). Thus far, most studies have focused mainly on leaves (Agarwal *et al.* 2006), but some have also focused on flowers (Rzanny *et al.* 2019, Seeland *et al.* 2019). By using artificial intelligence to detect plants, species identification can be performed by the general public. Because there is a shortage of taxonomic experts currently (Kim and Byrne 2006), biodiversity assessments can be conducted more effectively if plant species identification becomes easier via automation. In fact, applications have been developed to identify plant species in the field (Pärtel *et al.* 2021). The detection of exotic plants has also been conducted in the field. However, most existing research has been performed using satellite imagery and UAVs, and few examples of detecting specific exotic plants from action cameras have been reported in the relevant literature.

The purpose of this study was to identify flowers of the *Solidago altissima* (tall goldenrod), which were chosen as one of Japan’s worst 100 invasive species, from images captured using a small video camera, through deep learning. The challenges posed by this study are (1) whether it is possible to obtain image data that can be automatically identified using deep learning from commercially available small cameras, and (2) whether the target species can be effectively identified from a wide variety of objects in the image data. Having achieved these goals enabled us to monitor a wide range of areas, thus significantly improving future measures against invasive species.

## Materials and Methods

### S. altissima

*S. altissima*, is a rhizomatous perennial herb of the Asteraceae family, native to North America. It was chosen as one of Japan’s 100 worst invasive species, by the Ecological Society of Japan. The *S. altissima* was originally introduced as an ornamental plant, but is now commonly found throughout Japan, owing to its strong reproductive potential. It grows in open disturbed areas and agricultural fields, and its allelopathic effect inhibits the growth of other plant species, which is why it grows in dense clusters. In Japan, the growing season is March–October, and the flowering season is October–November; during which period, many small, dark yellow flowers appear.

### Study area and image acquisition

**Fig. 1.**
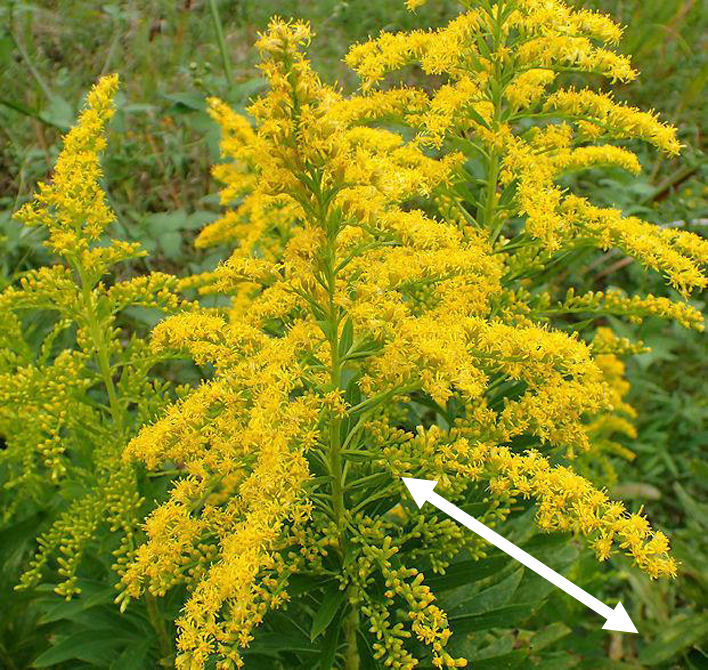
The primary inflorescence branch of *S. altissima* is used as a reference for measurement. The size of the training image was classified according to the number of pixels of the white arrow in the picture.

**Fig. 2.**
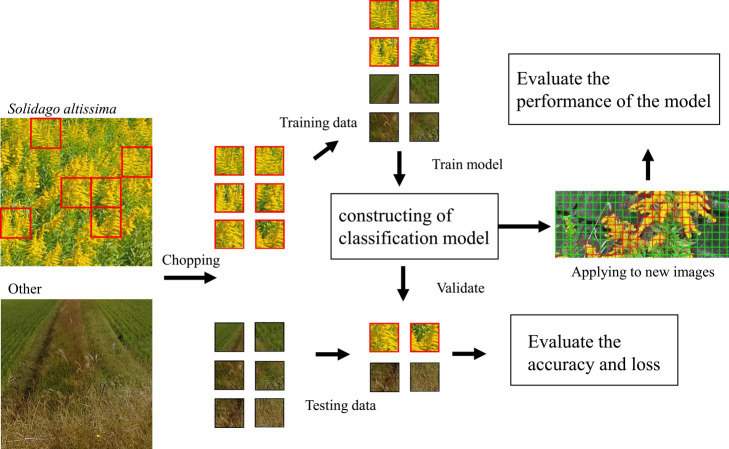
Overview of the approach adopted in this research. As positive data, only the flowering parts of *S. altissima* were prepared. After cutting an image of *S. altissima* produces a large number of squares. Only images where most of the squares were *S. altissima* were selected as positive images. The majority of the squares contained leaves that were removed, but some leaves were included in the positive data.

Two small video cameras (action cameras), a GoPro HERO 9, and a DJI Pocket2 were used to photograph the *S. altissima*. A mirrorless camera (OLYMPUS, OM-D E-M1 Mark II) was used to compare the data. The date, location, and image size of each camera are shown in Table 1, in which, GoPro HERO 9 and DJI Pocket2 were set to shoot videos at maximum resolution and converted to images using the Free Video to JPG Converter. The training images were created based on the number of pixels as follows: First, the primary inflorescence branch was defined as a part of the inflorescence that broke off from the inflorescence axis, and the number of pixels was measured (Fig. 1). The size of the primary inflorescence branch was then classified into three classes: S (10–50 pixels), M (51–100 pixels), and L (101–300 pixels). Further, the training images were created using the chopped picture method (Ise *et al.* 2018). This technique enables efficient automatic identification of vegetation by dividing the image into numerous small squares, which enables the identification of irregularly shaped objects, such as plant coverage. This approach has been used in previous research such as identification of moss species from digital camera images and bamboo coverage from Google earth images (Ise *et al.* 2018, Watanabe *et al.* 2020). Image identification using deep learning, such as convolutional neural network (CNN), requires that the shape of the object be in a similar form. However, vegetation coverages are often highly amorphous, and the usage of conventional methods such as CNN is not reasonable. Semantic segmentation can be used for amorphous objects, but preparation of training images for this method is highly labor-intensive. The chopped picture method enables us to identify vegetation coverage with irregular shapes, and also improves the efficiency of preparing training images. If this cost-effective method is used to distinguish specific invasive plants, it is expected to enable the implementation of effective invasive species countermeasures. In this study, we first prepared two images, one containing the yellow flowers of *S. altissima* (positive), and the other without *S. altissima* (negative). The images were then chopped into small 30-pixel squares that overlapped 50% horizontally and vertically (Supplemental Code 1). The images used and the outline of the approach are shown in Fig. 2.

### Dataset

In this study, two models were created for each camera. We created a model using images captured by each camera for both, positive and negative images. This model is denoted by Model 1. However, many false positives exist in Model 1; for example, leaves and yellow flowers other than *S. altissima* were detected as *S. altissima*. Therefore, we collected 85 objects from all camera images detected as false positives and added these images to negative images to reduce false negatives. This model is denoted by Model 2. To create each dataset, images classified into S, M, and L sizes were mixed and randomly extracted to include training images of each size. The details of each dataset are listed in Table 2. When we created positive data, we selected 30-pixel squares that are mostly covered by yellow flowers. Still, some of the positive data include a small part of green leaves. However, the percentage of green leaves was kept under 20% by manual removal of inappropriate images.

### Data augmentation

The chosen data augmentation techniques were wide shift, horizontal shift, horizontal flipping, and image zooming. Data augmentation can expand the training data and prevent overfitting. Because the pixel intensities of the image data were in the range 0–255, we normalized the data to an intensity between 0 and 1, by dividing by 255. Normalization was applied to the training and validation data.

### Neural network

**Fig. 3.**
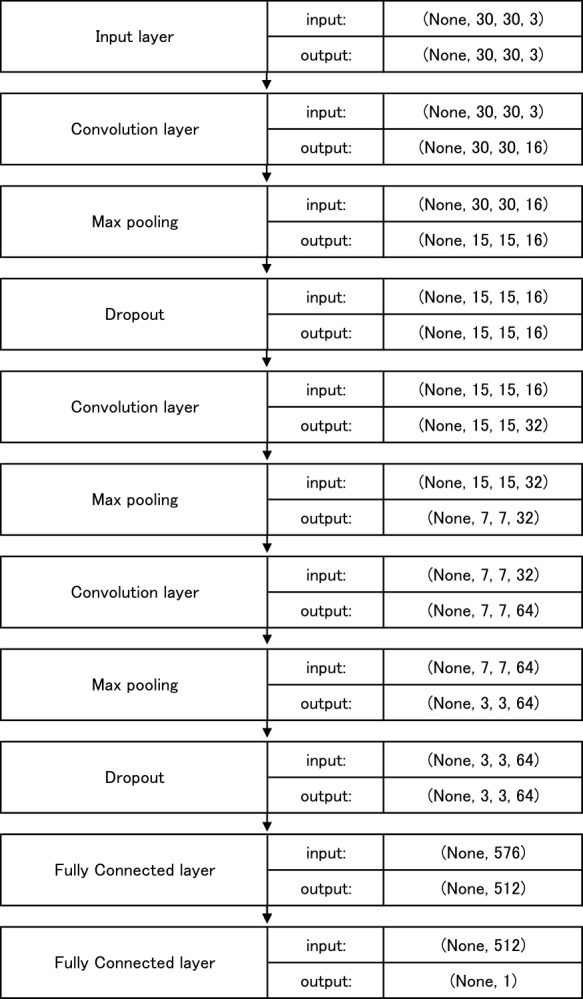
Architecture of the network used in this study.

In this study, we used a convolutional neural network (CNN) to identify *S. altissima*. A CNN is a neural network that consists of a convolution layer and a pooling layer. We used Keras, a deep learning framework, for the implementation of CNN. The parameters of the model are listed in Table 3, the network used is shown in Fig. 3. In convolution layer, zero padding was implemented, such that the size of the output image remained constant. The activation function converted the input value into a different number while it outputs one neuron after the other. In this study, we used a rectified linear unit (ReLU) as the activation function of convolution layer. This function outputs 0 when the input value is less than or equal to 0; and a value equivalent to the input value when the input value is greater than 0. It has become a standard activation function for deep neural networks because it tackles the problem of the vanishing gradient, and improves learning speed. The activation function for the final output of the model was sigmoid because the identification of *S. altissima* is a two-class classification. The optimization was based on Adam, and binary cross-entropy was used as the loss function. ResNet (He *et al.* 2016) and EfficientNet (Tan and Le 2019) have been proposed in recent years and have shown high identification accuracy. However, because we used small images as training and validation data in this study, we constructed our network with shallow network layers.

### Accuracy assessment

Some of previous studies created ground truth manually to measure the performance of automatic detection. In this study, we evaluated the classification performance of our model using the method reported by Watanabe *et al.* (2020). This is because the flower of *S. altissima* is too small and dense to create the ground truth. Furthermore, the chopped picture method does not assume pixel-level discrimination, contrary to the semantic segmentation method. However, although semantic segmentation can identify objects with high accuracy, it requires considerable effort to create a deep learning model. The chopped picture method can significantly reduce the effort required to create training data compared to conventional methods. In addition, performing pixel-level identification using the chopped picture method is difficult; however, the percentage of objects in an image can be estimated using this method, which can facilitate tasks such as invasive plant control. First, we selected the images obtained from three different cameras that were not used for training. From these images, we collected the ones that were nearly 100% covered by *S. altissima*, and not covered by *S. altissima*. Next, the randomly selected images were chopped into pieces of 30 pixels with 50% overlap in height and width, and 500 units of test data were obtained. Finally, each model identified test data that contained *S. altissima*, and those that did not contain *S. altissima*. We further counted the number of images identified as *S. altissima* and not *S. altissima*. The number of images identified as true positive (TP), false negative (FN), false positive (FP), and true negative (TN) were calculated. The same process was applied to all models, and the following equations were used to calculate each index: Accuracy = (TP + TN)/(TP + TN + FP + FN), Recall rate = TP/(TP + FN), Precision rate = TP/(TP + FP). The kappa coefficient (Cohen’s kappa) was also calculated to evaluate the model (Cohen 1960).

### Transferability test

*S. altissima* is established in diverse environments such as riparian areas, farmlands, and urban areas. In addition, in the application of this technology in citizen science, various camera hardware would be expected to be used by citizens based on their availability. Therefore, it is essential to determine whether a model created with images captured by a particular camera can identify images captured by other cameras. Hence, after creating a model for each camera, we conducted an accuracy assessment using an image captured with a different camera as a test image.

## Results

### Variation in accuracy and loss of learning

**Fig. 4.**
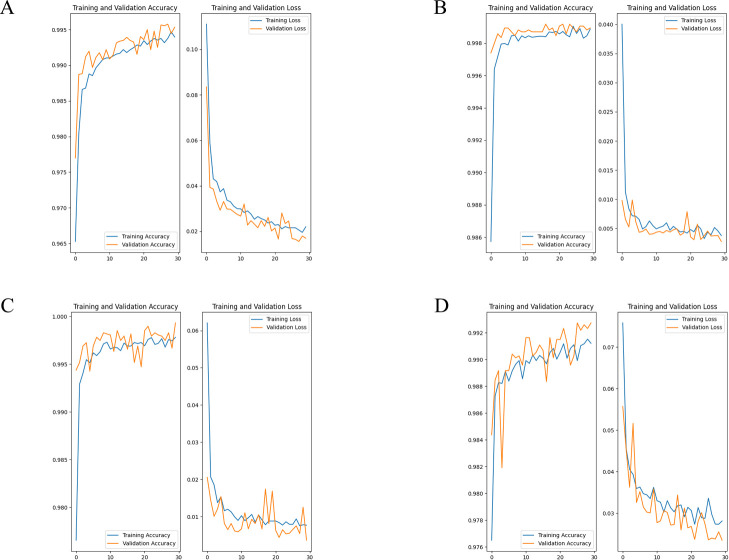
Validation accuracy and loss in model1. (A) OLYMPUS, OM-D E-M1 Mark II, (B) GoPro HERO 9, (C) DJI Pocket2, (D) All.

**Fig. 5.**
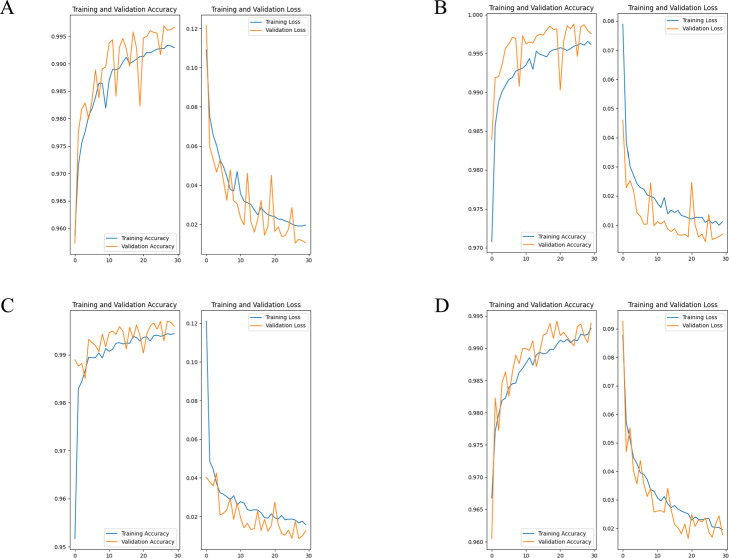
Validation accuracy and loss in model2. (A) OLYMPUS, OM-D E-M1 Mark II, (B) GoPro HERO 9, (C) DJI Pocket2, (D) All.

In Model 1, the validation accuracy ranged between 99.3% to 99.9%, with a mean of 99.7%. Validation loss ranged between 0.003 to 0.024, with a mean of 0.012 (Fig. 4). In Model 2, the validation accuracy ranged between 99.4% to 99.8%, with a mean of 99.6%. Validation loss ranged between 0.007 to 0.018, with a mean of 0.012 (Fig. 5). In all models, it was observed that the accuracy increased and the loss decreased, which suggests that the models were able to effectively learn the features of *S. altissima* and did not overfit the datasets.

### Performance of the model for each camera

**Fig. 6.**
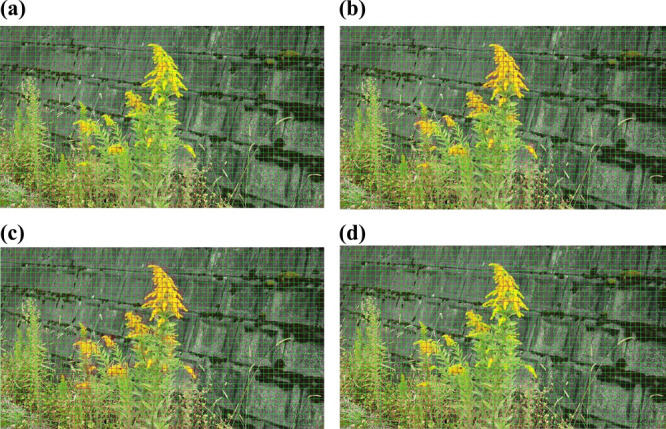
The results of Model 1 for the identification of *S. altissima*. All of the models identified *S. altissima*. (a) OLYMPUS, OM-D E-M1 Mark II, (b) GoPro HERO 9, (c) DJI Pocket2, (d) All. The red cells indicate that the model identified *S. altissima*, and the green cells indicate that the model identified something other than *S. altissima*.

**Fig. 7.**
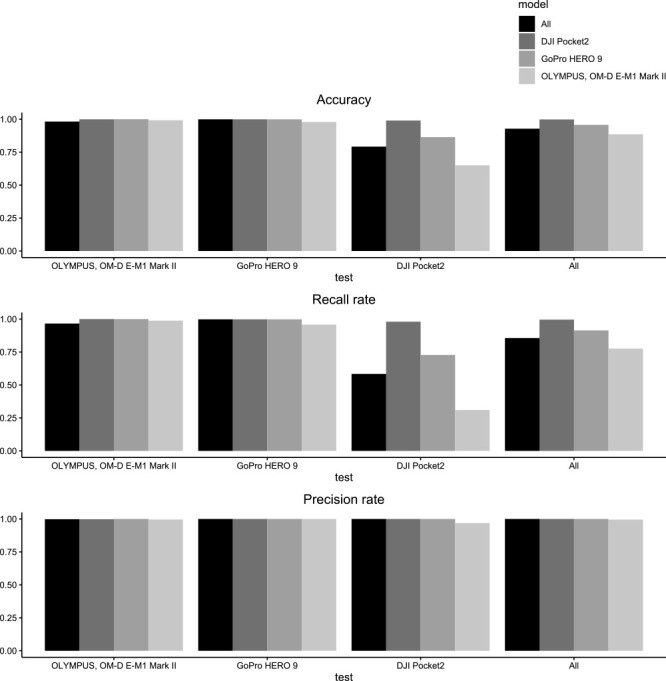
Comparison of classification performance in Model 1.

**Fig. 8.**
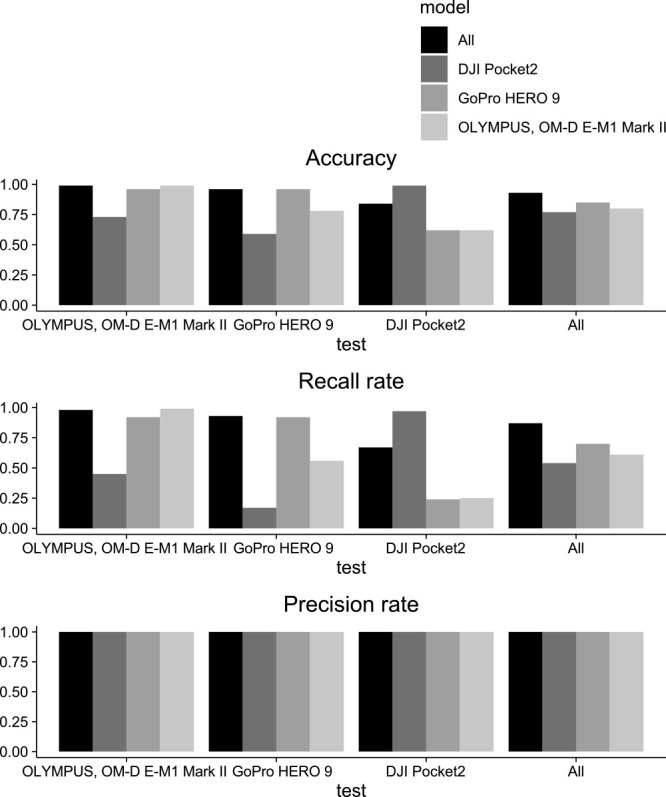
Comparison of classification performance in Model 2.

In Model 1, all models were able to identify *S. altissima*, as shown in Fig. 6. The classification accuracy for each camera in Model 1 ranged between 65% to 100%, with an average of 94% (Fig. 7). The kappa values ranged from 0.30 to 1.00, as displayed in Table 4. The classification accuracy for each camera in Model 2 ranged between 59% to 99%, with an average of 84% (Fig. 8). The kappa values ranged from 0.17 to 0.99, as listed in Table 5. The average classification accuracy for Model 1 and Model 2 was 89%. Models trained with only datasets of sizes S, M, and L were created, and the performance of these models is shown as Supplemental Table 1.

### Transferability of the model

**Fig. 9.**
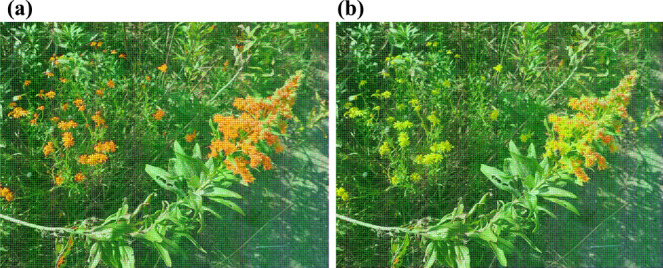
Results of yellow flower identification in DJI model. (a) Before adding yellow flowers to the negative images in Model 1. (b) After adding the yellow flowers to the negative images in Model 2.

**Fig. 10.**
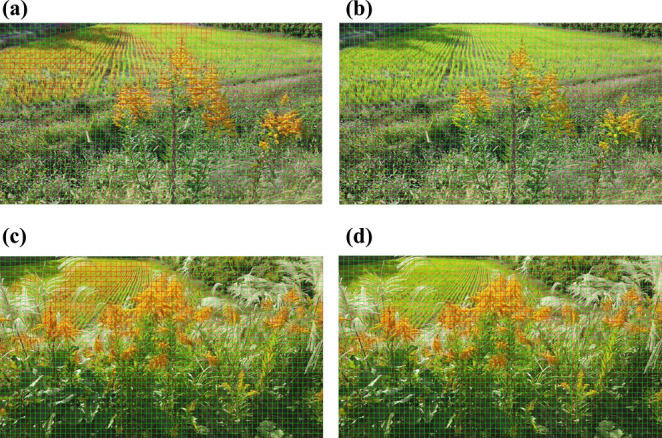
Main false positives. In Model 1, false positives were found on leaves exposed to strong sunlight, but in Model 2, false positives were reduced. The figure shows the results of identification in the all model, with (a) and (c) showing the results for Model 1 and (b) and (d) for Model 2.

**Fig. 11.**
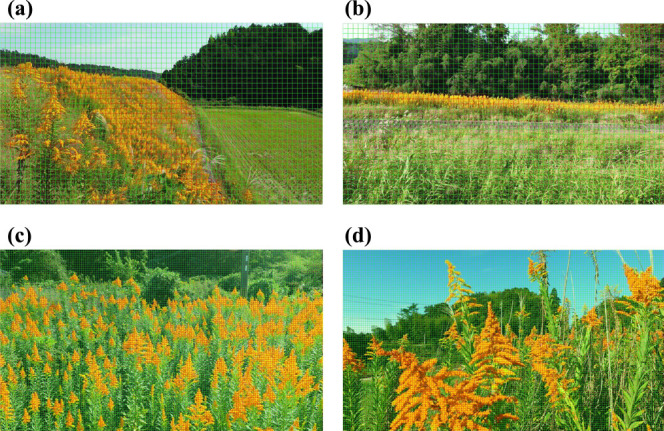
Results of identifying the test images with the all model in Model 2. (a) and (b) show images in which the *S. altissima* is small. (c) and (d) show images in which the *S. altissima* is large.

**Fig. 12.**
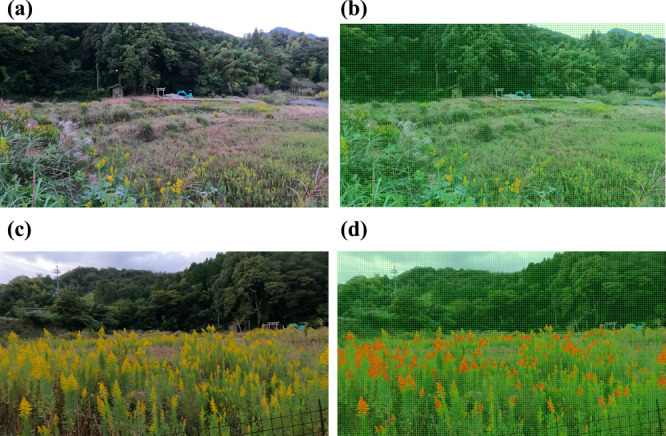
Results of identifying the cloudy images. (a) and (c) show image before identification. (b) and (d) show results of identification. Because the image was taken in the evening, the appearance of the image differs from other images.

In Model 1, the performance of the model using mirrorless camera images for the test data was superior (Fig. 7). The accuracy of said model in identifying the DJI test data was significantly lower; however, the transferability of the other models was high. Additionally, there were false positives for yellow flowers and leaves exposed to strong sunlight (Figs. 9, 10). For Model 2, false positives were reduced, and kappa values were higher when the model and test images were the same (Supplemental Fig. 1). Models captured with DJI images were able to identify other models; however, GoPro and mirrorless camera models were not able to identify DJI models. The model with a mixture of all images was able to identify all the *S. altissima* in each camera (Fig. 11). However, the cloudy images did not facilitate the identification of the *S. altissima* (Fig. 12).

## Discussion

In this study, we have demonstrated the identification of *S. altissima* using action camera images using the chopped picture method. Recently satellite images and UAVs have been used to detect invasive alien plants. However, the low resolution of satellite images and the flight restrictions on UAVs have posed an issue. On the other hand, the action camera has a high resolution and can acquire images in urban areas where UAVs are often restricted. Model 1 identified *S. altissima* with an average accuracy of 94%, while Model 2 demonstrated an average accuracy of 84%. It suggests that effective identification models can be prepared using inexpensive cameras, and it may open possibilities for citizen science in this field.

Image recognition techniques have been used for plant disease and species identification (Pärtel *et al.* 2021, Singh *et al.* 2018). However, few studies have used deep learning for detecting alien plant species. Moreover, most of those studies used UAVs (James and Bradshaw 2020, Qian *et al.* 2020), and few studies combined it with action cameras for plant species identification. The use of action cameras will enable the automatic identification of invasive species even in areas where UAV flights are difficult. In addition, pixel-based and object-based image analysis have been used in previous studies for automated image classification (Dash *et al.* 2019). In contrast to these methods, the chopped picture method facilitates the acquisition of training images, allowing us to create a specific model for each region. Since the established alien species vary from region to region, this method creates region-specific alien species identification models.

The results were high in accuracy and low in recall rate, suggesting that the model used in this study performed conservative classification with a relatively high number of false negatives. Comparing the results of each model, the kappa value was higher when the mirrorless camera was used for the test image. Because the resolution of the mirrorless camera was the highest among the cameras used in this study, the image resolution had a significant impact on the performance. On the other hand, the model created with DJI images, which had the lowest resolution, had the lowest kappa value, suggesting that a higher resolution camera would be preferable to identify *S. altissima*. We have created a new model for each size, and the performance of these models is now shown as a Supplemental Table 1. By comparing the results, we found that the performance of size-specialized models was better than the general model in the main manuscript, indicating that the size separation can improve the performance. However, variations and uncertainties in the pictures (e.g., light conditions) were present and the comparison was somewhat vague. Since these factors may have affected the results, the performance evaluation should be conducted under uniform conditions in the future.

For the model transferability, the kappa value was high when the images used for the training data of the model and those used for performance evaluation, were the same. Previous studies have also proved that the conditions at the local scale of the training data affect the classification accuracy (Watanabe *et al.* 2020). Therefore, to create a high-performance CNN model, it is necessary to create training images based on the region. Because the chopped picture method simplifies the creation of training images, it enables the construction of CNN models suitable for specific region with low effort. Furthermore, it is an effective method for detecting invasive alien plants, such as *S. altissima* in a wide area.

The reason why all models were able to identify the *S. altissima* was probably the color of the flowers. Because this species has bright yellow flowers, it can easily be distinguished from surrounding objects. Another reason is that there were no other plants with yellow flowers in the images used for identification. In this study, we focused on flowers that were easy to identify using deep learning. Although plant identification is difficult for non-specialists, it is easy to distinguish flowers from other species in the field. There are many cases in which herbaceous plants introduced for ornamental purposes became invasive species, and by focusing on flowers, these plants are easy to identify with high accuracy. Furthermore, in a previous study, the number of flowers was measured by deep learning (Palacios *et al.* 2020), so it may be possible to identify factors that influence the expansion of the distribution of alien species by clarifying the number of flowers and individuals using deep learning, in the future.

In this study, we obtained all images on the same day in geographically similar locations, and the weather of the day was mostly sunny. These facts may positively affect the accuracy of object identification by deep learning. To make the model robust for citizen science, in the future, images captured under various environmental conditions such as location and weather should be added to the training images.

In addition, it was possible to identify the flowers regardless of their size. This may also be because training images were created and trained for each flower size. Another reason may be that the color of the flowers is distinguishable from their surroundings. In Japan, there are other non-native species with yellow flowers, such as *Coreopsis lanceolata*. Because automatic identification of non-native plants with yellow flowers is relatively easy, it is necessary to investigate whether this method can be applied to other non-native plants in the future.

In the present model, there were a few false positives for the sky and mountains. It is assumed that the sky and mountains were easily distinguishable, were included in the images captured by all the cameras, and that there were enough images in the training images. However, there were false positives for leaves and stems that were strongly exposed to sunlight. When the sunlight is strong, it is assumed that it may be difficult to distinguish yellow flowers from green leaves and stems. This may have been a result of the training image used in this study being exposed to intense sunlight. In addition, the performance of the model was improved by adding false-positive images to the training images; hence, the lack of variation in the training images may also be a contributor. Although *S. altissima* flowers have the same color in all regions, the backgrounds of the captured images vary. Therefore, it is essential to prepare negative images based on the region to create a model for identifying *S. altissima* in each area.

It has been difficult for deep learning to identify irregularly shaped objects such as plants. However, by utilizing the chopped picture method, a wide range of plants can be identified. This method can also be applied in plant breeding for selecting individuals with beneficial traits and for effective daily management. For example, by identifying the timing of flowering or fruition, proper management can be achieved. It may also be possible to identify individuals with the best traits for breeding.

Owing to their growing popularity worldwide, small, high-performance video cameras can be purchased at low prices. Small video cameras that are lightweight, durable, and waterproof can capture high-resolution images; they have long battery life and can be easily used in the field, as they have begun to be used in the field of ecology (Claassens and Hodgson 2018, Gilpin *et al.* 2017). Various methods have been devised to detect invasive alien species in the early stages of their invasion, and combining small cameras with deep learning may become a new method for the detection of alien species. In particular, the widespread use of smartphones makes it easier to take photos, even outdoors. In the future, citizen science will be used to understand the distribution of invasive alien species over a wide area, using deep learning to analyze a vast number of images obtained from the citizens.

In this study, we created a model that can identify the invasive alien plant species, *S. altissima*, by combining the chopped picture method and deep learning. As the model was able to identify the invasive species from images taken with an ordinary camera, it may become a valuable method in citizen science, for understanding the distribution of invasive species. Because deep learning makes it possible to analyze large amounts of data, it enables research on a scale that was previously difficult to analyze. For example, drive recorders are being extensively used, and an environment is being created in which images of the area around roads can be easily acquired. If alien plants can be identified from the images of citizens’ drive recorders, we can also receive data from people who are not consciously participating in citizen science. Privacy issues can be avoided by processing the data provided using deep learning, rather than by humans. Furthermore, once we create a system to share drive recorder images in real-time, we will be able to understand the distribution of invasive species while updating the data at a high frequency. By further improving the model and overcoming technical issues, it is possible to realize this innovative invasive species management system in the future.

## Author Contribution Statement

KT performed experiments and wrote the manuscript. YS performed experiments. TI guided all steps of the experiments and manuscript preparation.

## Supplementary Material

Supplemental Code

Supplemental Figure

Supplemental Table

## Figures and Tables

**Table 1.** Camera, date, location, and image size in this study

Camera	Date	Location	Image size (pix)
OLYMPUS, OM-D E-M1 Mark II	October 2020	Kyoto Shiga	5184 × 3888
GoPro HERO 9	October 2020	Kyoto	5120 × 2880
DJI Pocket2	October 2020	Shiga	1920 × 1080

**Table 2.** Number of training images

Model name	Camera	Original images		Chopped images	
		*S. altissima*	Other	*S. altissima*	Other
Model 1	OLYMPUS, OM-D E-M1 Mark II	7	30	15374	30720
	GoPro HERO 9	26	30	11520	30720
	DJI Pocket2	209	30	15216	28777
	All	70	29	7416	28744
Model 2	OLYMPUS, OM-D E-M1 Mark II	7	115	15374	62349
	GoPro HERO 9	26	115	11520	62349
	DJI Pocket2	209	115	15216	62349
	All	70	115	7416	62349

**Table 3.** Parameter settings

Settings	Selected options
Training epochs	30
Batch size	128
Dropout	0.2
Learning rate	0.001

**Table 4.** The kappa value in Model 1

Model	Test data			
	OLYMPUS, OM-D E-M1 Mark II	GoPro HERO 9	DJI Pocket2	All
OLYMPUS, OM-D E-M1 Mark II	0.98	0.96	0.30	0.77
GoPro HERO 9	1.00	1.00	0.73	0.91
DJI Pocket2	1.00	1.00	0.98	1.00
All	0.96	1.00	0.58	0.86
Average	0.99	0.99	0.65	0.89

**Table 5.** The kappa value in Model 2

Model	Test data			
	OLYMPUS, OM-D E-M1 Mark II	GoPro HERO 9	DJI Pocket2	All
OLYMPUS, OM-D E-M1 Mark II	0.99	0.56	0.25	0.61
GoPro HERO 9	0.92	0.92	0.24	0.70
DJI Pocket2	0.45	0.17	0.97	0.54
All	0.98	0.93	0.67	0.87
Average	0.84	0.65	0.53	0.68
