# Supplementary material for: Automatic detection of alien plant species in action camera images using the chopped picture method and the potential of citizen science
Source: Breed Sci. 2022 Feb 5;72(1):96–106. doi: 10.1270/jsbbs.21062 (PMC8987844; doi:10.1270/jsbbs.21062)
Supplement: Supplementary file 1 — Supplemental Code [file 72_096_s1.pdf]

doi: 10.1270/jsbbs.21062

Supplemental Code 1.

```
###### initial settings ####################################


type <- ""
train_img_dir <- "/home/ise/deep/pics/training_image" # train img dir

### parameters ###
siz  <- 30 # chopping size (pix)
ovr <- 0.5 # overlap
fwd  <- siz*(1-ovr) ## window move size
#######################################################


library(png) # read R package. Before use this code, user have to intall "png
package" into R.
library(jpeg) # read R package. Before use this code, user have to intall "jpeg
package" into R.

### initialize dir
dir_chop_image <- paste(train_img_dir,"chopped",type, sep = "/")
setwd(dir_chop_image)
setwd("../")

if (file.exists(paste(type))){
    setwd(dir_chop_image)
     file.remove(list.files())
     setwd("../")
     file.remove(paste(type))
    }

dir.create(dir_chop_image)

### read train img
dir_image <- paste(train_img_dir,"original",type, sep = "/")
setwd(dir_image)
picList <- list.files(pattern='.png')

### chopped picture method
for (i in 1:length(picList)) {
    title <- picList[i]
    pic <- readPNG(title)
    dim(pic)
    xlen <- dim(pic)[[1]]
    ylen <- dim(pic)[[2]]
    xnum <- floor(xlen/fwd)-1
    ynum <- floor(ylen/fwd)-1
    count <-1
    setwd(dir_chop_image)
        for(x in 1:xnum){
            for(y in 1:ynum){
                small  <-
pic[((x-1)*fwd+1):(((x-1)*fwd+1)+siz-1),((y-1)*fwd+1):(((y-1)*fwd+1)+siz-1),]
                picname <- title <- gsub("¥¥.[0-9A-Za-z]+$", "", title)
                newname <- paste0(picname,"_",count,".jpg")
                writeJPEG(small,target = newname,quality=1)
                count <- count + 1
```

```
                }
            }
    setwd(dir_image)
}
```
