# Supplementary material for: Automatic detection of alien plant species in action camera images using the chopped picture method and the potential of citizen science
Source: Breed Sci. 2022 Feb 5;72(1):96–106. doi: 10.1270/jsbbs.21062 (PMC8987844; doi:10.1270/jsbbs.21062)
Supplement: Supplementary file 2 — Supplemental Figure [file 72_096_s2.pdf]

doi: 10.1270/jsbbs.21062

(a) (b)

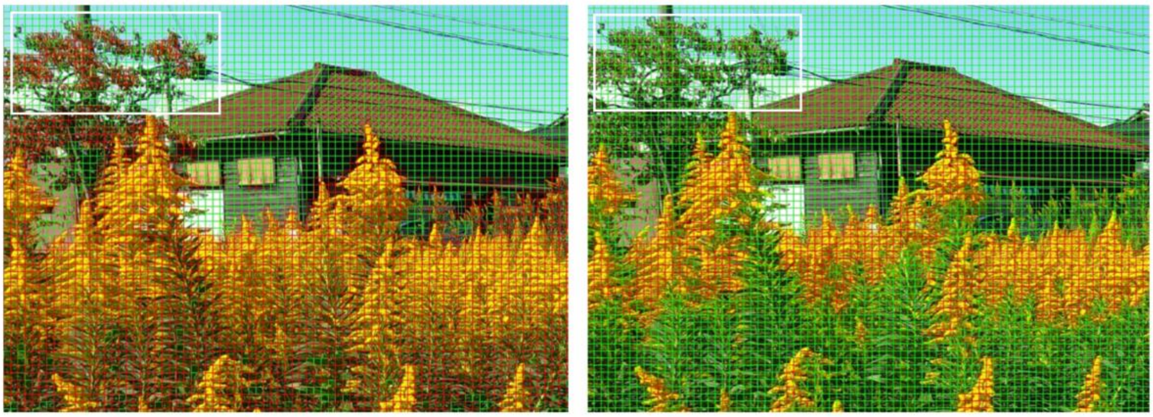

Supplemental Fig. 1. Results of identification in the Gopro model. (a) Before adding negative images in model 1. (b) After adding the negative images in model 2. False positive reactions for the leaves in the white boxes are reduced by adding negative images. The leaves of *S. altissima* were detected as false negatives in model 1, but these reactions were also reduced in model 2.
