# Supplementary material for: Automatic detection of alien plant species in action camera images using the chopped picture method and the potential of citizen science
Source: Breed Sci. 2022 Feb 5;72(1):96–106. doi: 10.1270/jsbbs.21062 (PMC8987844; doi:10.1270/jsbbs.21062)
Supplement: Supplementary file 3 — Supplemental Table [file 72_096_s3.pdf]

doi: 10.1270/jsbbs.21062

Supplemental Table 1. Comparison of the Kappa values between each size model and Model 2. The test data was a mixture of images from all cameras

	test data (All)			
model	S size	M size	L size	model2
OLYMPUS, OM-D E-M1 Mark II	0.82	0.86	0.8	0.61
GoPro HERO 9	0.9	0.88	0.87	0.7
DJI Pocket2	0.99	0.99	0.99	0.54
All	1	0.99	0.95	0.87
